# Ein klinisch-radiologischer Score für Femurkopftransplantate: Etablierung des Tabea-FK-Scores zur Sicherung der Qualität humaner Femurkopftransplantate

**DOI:** 10.1007/s00132-020-03941-5

**Published:** 2020-07-08

**Authors:** Nicolaus Siemssen, Christian Friesecke, Christine Wolff, Gisela Beller, Katharina Wassilew, Bruno Neuner, Helge Schönfeld, Axel Pruß

**Affiliations:** 1 Abteilung für Endoprothetik und Gelenkchirurgie, Krankenhaus Tabea GmbH & Co. KG, Kösterbergstr. 32, 22587 Hamburg, Deutschland; 2 Zentrum für Muskel- und Knochenforschung, Charité – Universitätsmedizin Berlin, CBF, Hindenburgdamm 30, 12203 Berlin, Deutschland; 3 Fachbereich Pathologie, Deutsches Herzzentrum Berlin (DHZB), Augustenburger Platz 1, 13353 Berlin, Deutschland; 4 Klinik für Anästhesiologie m.S. operative Intensivmedizin, Charité – Universitätsmedizin Berlin, CCM, Charitéplatz 1, 10117 Berlin, Deutschland; 5 Univ.-Gewebebank, Institut für Transfusionsmedizin, Charité – Universitätsmedizin Berlin, CCM, Charitéplatz 1, 10117 Berlin, Deutschland; 6 Institut für Laboratoriumsmedizin, Klinische Chemie und Pathobiochemie, Charité – Universitätsmedizin Berlin, CVK, Augustenburger Platz 1, 13353 Berlin, Deutschland

**Keywords:** Knochenbank, Knochentransplantation, Spongiosa, Femur, Gewebebank, Bone banks, Bone transplantation, Cancellous bone, Femur, Tissue banks

## Abstract

**Einleitung:**

Die Transplantation humaner Femurköpfe (FK) ist ein etabliertes Verfahren in der knöchernen Defektrekonstruktion bei orthopädischen und unfallchirurgischen Eingriffen, wobei eine standardisierte morphologische Qualitätsbewertung des Femurkopfgewebes bisher kaum erfolgt.

**Material und Methoden:**

Bei 105 Patienten wurde der jeweilige FK im Rahmen einer Hüft-TEP standardisiert entnommen. Anhand klinischer und radiologischer Kriterien (Vorhandensein von Zysten, Nekrosen, Verkalkungen, Deformitäten und Osteoporose) wurde der FK mittels des Tabea-FK-Scores in drei Kategorien (beste/mittlere/schlechte Qualität) eingeteilt. Hiernach erfolgte eine zweite, makroskopische Einteilung der Qualität des in drei Schichten aufgesägten FK. Dieser „Makro-Score“ diente als Goldstandard. Ergänzend wurden eine periphere quantitative Computertomographie (pQCT) sowie histologische Untersuchungen durchgeführt. Die Übereinstimmung des Tabea-FK-Scores sowie der Ergebnisse der ergänzenden Untersuchungen mit dem Makro-Score wurde mittels Sensitivitäten und Spezifitäten beurteilt.

**Ergebnisse:**

Bei 91/105 Patienten (Alter: 68,4 ± 9,9 Jahre, *n* = 60 Frauen, *n* = 31 Männer) wurden die explantierten FK in die Studie eingeschlossen. Die Übereinstimmung zwischen dem primären Tabea-FK-Score und dem Makro-Score in Bezug auf die Unterscheidung mittlere/beste sowie schlechte/mittlere Qualität ist als gut einzustufen (Sensitivität 77 % sowie 81 % und Spezifität 76 % sowie 84 %). Die Übereinstimmung des histologischen Befundes mit dem Makro-Score war insgesamt etwas schlechter und wies in Bezug auf die Unterscheidung mittlere/beste sowie schlechte/mittlere Qualität eine Sensitivität von 85 % sowie 54 % und eine Spezifität von 66 % sowie 97 % auf. Der pQCT-Score wies lediglich bei der Unterscheidung mittlere/beste Qualität eine zufriedenstellende Sensitivität (82 %) auf, während diese bei der Unterscheidung schlechte/mittlere Qualität sowie schlechte/mittlere + beste Qualität <10 % betrug.

**Diskussion:**

Die gute Übereinstimmung zwischen primärem Tabea-FK-Score und makroskopischer Zweitbewertung unterstreicht die Fähigkeiten langjährig operativ tätiger Orthopäden, die Qualität der Knochenspongiosa bereits anhand des Röntgenbildes und des intraoperativen Befundes korrekt einzustufen. Insofern kann die Einführung des Tabea-FK-Scores in die Routineabläufe von Knochenbanken als Qualitätssicherungsmaßnahme empfohlen werden.

## Abstract (en)

**Introduction:**

Transplantation of cancellous tissue from human femoral heads (FK) is an established method in the reconstruction of bony defects in orthopedic and trauma surgery. Standardized rating systems with respect to the morphological quality of this tissue are not available.

**Materials and methods:**

In 91/105 patients who had been a regular, clinically-indicated surgery (arthroplasty of the hip joint) the respective femoral head (FK) was taken under standardized conditions. Using a checklist defined clinical and radiological criteria of FK are judged in terms of their quality (cysts, necrosis, calcification, deformities, osteoporosis) and divided by the Tabea FK score into three classes (best/middle/poor quality). This was followed by a blinded repeated scoring, now as macroscopic assessment of three sawed layers from the same femoral head. The femoral heads are examined by peripheral quantitative computed tomography (pQCT) and a standardized histological examination of the bony tissue. We evaluated the accordance of the Tabea FK score with complementary assessments by calculation of sensitivity and specificity.

**Results:**

Femoral heads from 91/105 patients (ages: 68.4 ± 9.9 , *n* = 60 women, *n* = 31 men) were explanted and included in the study. The correlation between the primary radiologic clinical score (Tabea FK score) and the macroscopic second review of the sawn FK with respect to middle/best and poor/middle quality was classified as good (sensitivity 77% and 81%, respectively; specificity 76% and 84%, respectively). The correlation of histology and macroscopic second review was worse and in relation to discrimination of middle/best and poor/middle quality had a sensitivity of 85% and 54%, respectively, and a specificity of 66% and 97%, respectively. The pQCT showed a sensitivity of 82% only in discrimination of middle/best, while sensitivity in discrimination of poor/middle and poor/middle + best, respectively, was <10%.

**Discussion:**

The corresponding correlation between the primary and the second clinical score was evaluated as good. This emphasizes the long-standing skills of operationally active orthopedic surgeons to classify the quality of cancellous bone correctly already on the basis of X‑ray images and intraoperative findings. In this respect, the introduction of the Tabea FK score as a quality assurance tool in the routines of bone banks can be recommended.

Die Transplantation humaner Femurköpfe (FK) ist ein etabliertes Verfahren in der knöchernen Defektrekonstruktion im Rahmen orthopädisch-unfallchirurgischer Eingriffe. Neben lokalen Knochenbanken fungieren überregionale Anbieter als Lieferanten für FK, die in nach §20b AMG [2] berechtigten Entnahmeeinrichtungen gesammelt werden. Die Kriterien für die Eignung des Spenders sind im TPG [4] bzw. der TPG-GewV [3] festgelegt. Die Beurteilung, ob der FK für die Transplantation geeignet ist, trifft der entnehmende Arzt nach radiologischem und intraoperativem Eindruck.

Die Vergangenheit hat gezeigt, dass die gespendeten FK eine sehr große Varianz hinsichtlich Größe, spongiöser Dichte und degenerativem Knochenabbau zeigen. Zwar hat die Einführung des Single European Code (SEC) für eine Verbesserung der Transplantatdeklaration und der Traecability geführt, jedoch werden auch in diesem europäischen System keine klinischen Qualitätsparameter berücksichtigt [11, 18].

## Hintergrund und Fragestellung

Es existieren verschiedene, nichtkontrollierte klinische Studien mit thermodesinfizierten FK-Transplantaten, die sich vor allem mit der biomechanischen Stabilität des Knochengewebes, auch im Vergleich zu allogenen Knochenpräparaten, befassen. Die Studien liefern auch Erkenntnisse zur klinischen Sicherheit der FK als allogenes Transplantat. Siemssen [20] untersuchte den Operationserfolg nach allogener Knochentransplantation in der Revisionsalloarthroplastik des Hüftgelenks. Er analysierte retrospektiv 2 Patientenkollektive mit je 128 bzw. 108 Fällen und kam zu dem Schluss, dass kein Unterschied im Prozentsatz der nach 4–5 Jahren noch festsitzenden Prothesen erkennbar und damit kein Unterschied in der biomechanischen Stabilität und dem Einwachsen des Transplantates in den Empfängerknochen im Vergleich zum gammabestrahlten Präparat vorhanden ist. Die Infektionsrate von 1,6 % bzw. 1,8 % lag unter dem in der Literatur beschriebenen Wert von 3,5 %. Eine Bewertung der morphologischen Qualität des FK erfolgte nicht. Die prospektiv geplante Studie von T. von Garrel [25] erlaubte keine Aussage zur Wirksamkeit. Hier gab es laut Studienprotokoll eine „deskriptive Analyse“ der Daten, die aber nicht präsentiert wurden. Die Rate an Knocheninfektionen von 4,6 %, einschließlich transplantatbedingter Komplikationen, zeigte sich in dieser Studie als vergleichbar mit publizierten Daten von nichtbehandelten allogenen Knochentransplantaten. In der Studie von Junge et al. [8] wurden alle Transplantate als fest eingeheilt bewertet. In der Publikation gibt es zwar eine Aussage, dass keine Unterschiede in der Infektionsrate zwischen nur kryokonservierten und zusätzlich thermodesinfizierten Transplantaten festgestellt wurden, jedoch fehlen die Daten dazu. Tiefe Infekte wurden nicht beobachtet. Biopsien von 17 Patienten zeigten, dass das allogene Transplantat in den Knochen eingebaut wurde. Da es sich bei keiner der mit thermodesinfiziertem FK durchgeführten Studien um prospektiv geplante, kontrollierte Studien handelt, kann keine Aussage darüber getroffen werden, ob die Wirksamkeit und Sicherheit des allogenen Transplantates nach Thermodesinfektion vergleichbar oder besser ist als allogene Transplantate, die nach einem anderen Verfahren hergestellt wurden. Selbst die Untersuchung von Junge et al. [7] an einem relativ großen Patientenkollektiv erlaubt keine Aussage darüber, ob eine Überlegenheit der zusätzlichen Thermodesinfektion gegenüber einer alleinigen Kryokonservierung vorhanden ist, obwohl die alleinige Kryokonservierung wegen Sicherheitsbedenken aufgegeben wurde. Eine standardisierte Bewertung der morphologischen Qualität des FK erfolgte auch in diesen Studien nicht.

Somit ist festzuhalten, dass zwar eine Vielzahl von Daten für die Sicherheitsstrategie sowie die klinischen Einsatzgebiete von Femurkopftransplantaten vorliegen, jedoch keine wissenschaftlichen Untersuchungen bzw. geeignete Wertungssysteme bezüglich der morphologischen Qualität des Ausgangsmaterials und damit der Voraussetzung für den klinischen Transplantationserfolg vorliegen. Ziel der vorliegenden Studie war deshalb, einen im klinischen Alltag möglichst praktisch handhabbaren Score zu entwickeln. Dieser soll zuverlässig die Eignung des Kopfes für die Transplantation ermitteln. Die Bewertung soll anhand präoperativer radiologischer Kriterien möglich sein und intraoperativ klinisch überprüft werden. Aus der Bewertung soll eine Aussage hinsichtlich der Verwertbarkeit des FK als strukturelles oder nur „morcellized“ Graft möglich werden.

Die FK wurden in der vorliegenden Arbeit auch mittels peripherer quantitativer Computertomographie (pQCT) untersucht. Mit der pQCT ist man in der Lage, spongiösen von kortikalem Knochen zu differenzieren. Die Knochendichte, -masse und die Masseverteilung kann bildlich dargestellt werden und anteilsmäßig sowohl die Dichte (BMD, „bone mineral density“) als auch die Masse (BMC, „bone mineral content“) quantifiziert werden. Die pQCT könnte somit bei der Validierung von Qualitätskriterien zur Charakterisierung von Knochenfragmenten aus Spenderknochen genutzt werden. Schließlich erfolgte eine standardisierte histologische Untersuchung des Knochengewebes. Die Studie sollte auch klären, ob zusätzliche Untersuchungen wie pQCT und histologische Befunde zwingend zur Beurteilung der Knochenqualität erforderlich oder verzichtbar sind.

Im Ergebnis sollte der vorgeschlagene klinisch-radiologische Qualitätsscore auf seine Eignung für die Praxis untersucht werden.

## Studiendesign und Methoden

### Prä- und intraoperative Primäreinstufung (Tabea-FK-Score)

**Figure 1:**
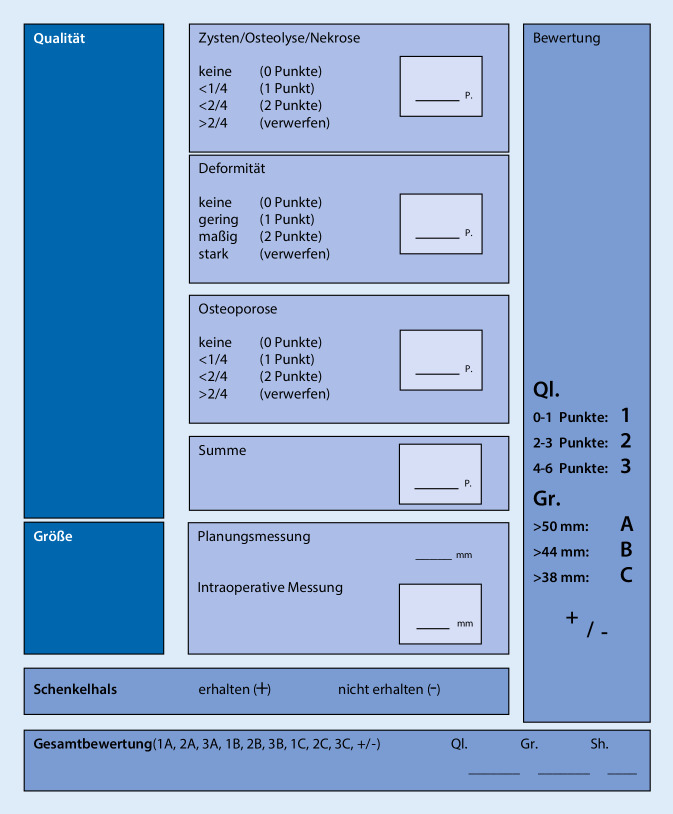

Bei 105 Patienten, die zu einer regulären, klinisch indizierten Operation (Totalendoprothese des Hüftgelenks) in das Krankenhaus Tabea, Hamburg, eingewiesen wurden, wurde die Zustimmung der Patienten zur Studie eingeholt und der jeweilige FK standardisiert entnommen. Bei 91/105 Patienten (Alter: 68,4 ± 9,9 Jahre, *n* = 60 Frauen (65,9 %), *n* = 31 Männer (34,1 %)) wurden die explantierten FK in die Studie eingeschlossen. Die Einschlusskriterien entsprachen denen der regulären, klinisch indizierten TEP-Operation des Hüftgelenks. Damit entfällt die Definition von Ausschlusskriterien. Eine Aussage über die Eignung und Art der Anwendung eines FK in der Transplantationschirurgie konnte zum Entnahmezeitpunkt nur anhand der präoperativen Bildgebung und intraoperativ nach klinischem Eindruck getroffen werden. Als geeignete Kriterien waren zum einen die Größe und Form, zum anderen die Qualität, respektive insgesamt die Masse zu beurteilen. Um eine reproduzierbare Bewertung zu gewährleisten, wurde eine Checkliste erarbeitet, mithilfe derer letztlich eine klinisch nützliche Gesamtbewertung abgegeben werden kann (Abb. 1). Die Größe wurde im Rahmen der EDV-gestützten Operationsplanung bestimmt und intraoperativ kontrolliert.

**Figure 2:**
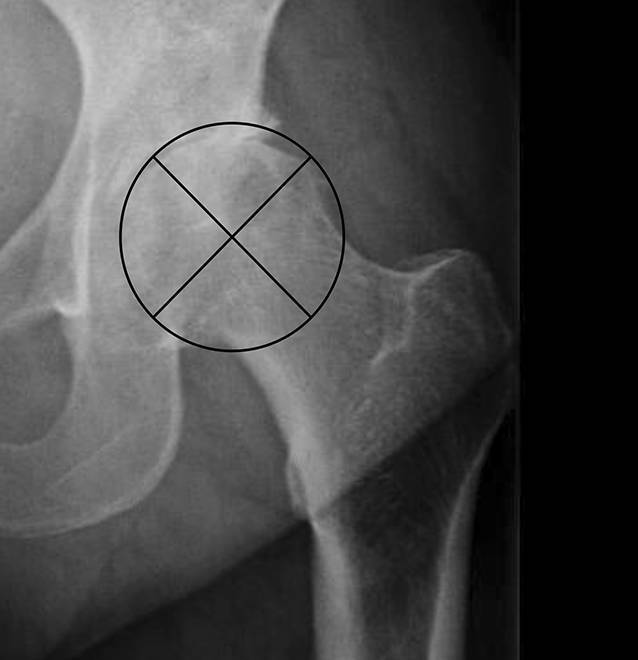

Die Qualität wurde in einem Punktesystem anhand von Kriterien bewertet, die sich aus dem präoperativen Röntgenbild bestimmen ließen. Hierbei war entscheidend, welcher Anteil des FK überhaupt zur Transplantation geeignet ist. Der FK wurde hier radiologisch in beiden Bildebenen geviertelt und zum einen der Anteil an nekrotischen/zystischen/osteolytischen Zonen, zum anderen der Anteil an deformitätsbedingtem Substanzverlust bestimmt (Abb. 2 und 3). Zusätzlich ging der intraoperative Eindruck der Knochendichte in der Sägeebene und präparierten azetabulären und femoralen Implantatlager mit in die Bewertung ein. Die klinische Beurteilung dieser Qualitäten ist wichtig, da für die meisten knöchernen Rekonstruktionen die spongiöse Knochendichte des FK einen großen Einfluss auf die Menge und insbesondere Stabilität hat.

**Figure 3:**
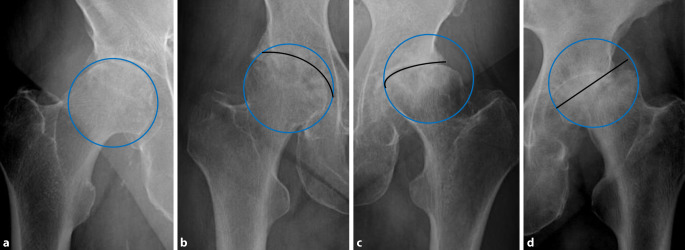

**Figure 4:**
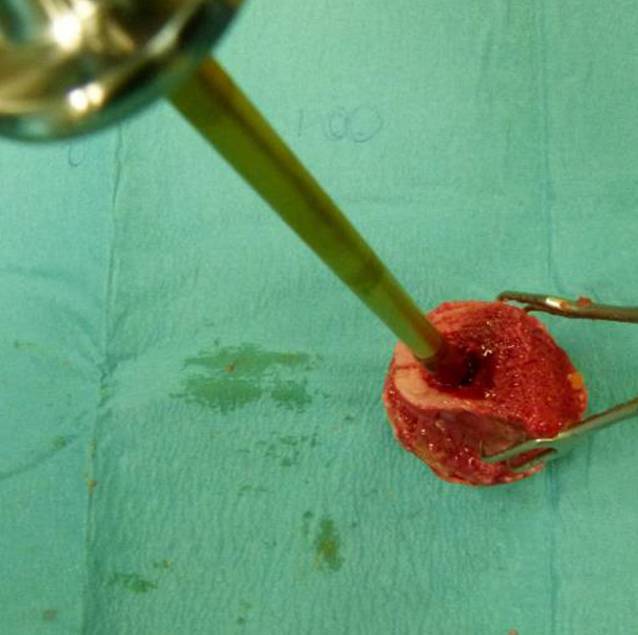

Der FK wurde in die Größenkategorien A (>50 mm), B (>44 mm) und C (>38 mm) eingestuft, orientiert in der Untergrenze an die minimale Größe der Thermodesinfektion. Die Einstufung der Qualität erfolgte in den Stufen 1–3. Somit kann in 9 Bewertungen (A1–3, B1–3 und C1–3) unterteilt werden. Als zusätzliches Merkmal wurde das Vorhandensein eines Schenkelhalses berücksichtigt, was bei der Auswahl für ein strukturiertes Transplantat klinisch relevant sein kann.

Der FK, der in der Checkliste bereits in einem einzelnen der Kriterien in die Kategorie „verwerfen“ einzuordnen ist, sollte nicht zur Transplantation freigegeben werden.

Die FK wurden anschließend tiefgefroren in das Institut für Transfusionsmedizin, Charité – Universitätsmedizin Berlin, Campus Charité Mitte, verbracht. Dies geschah in geeigneten Transportgefäßen (Fa. Telos, Marburg, Deutschland) unter kontrollierten Bedingungen.

### Periphere quantitative Computertomographie

Die FK wurden im Zentrum für Muskel- und Knochenforschung am Campus Benjamin Franklin der Charité mittels pQCT untersucht. Mit der pQCT ist es möglich, spongiösen von kortikalem Knochen zu differenzieren. Die Knochendichte, -masse und die Masseverteilung kann bildlich dargestellt werden und anteilsmäßig sowohl die Dichte (BMD, „bone mineral density“), als auch die Masse (BMC, „bone mineral content“) und Querschnittsflächen (CSA) quantifiziert werden. Die pQCT-Untersuchungen erfolgten mittels einem XCT 2000L, Modell 2002 (Stratec Medizintechnik, Pforzheim, Deutschland). Die Auflösung beträgt 0,5 × 0,5 mm, die Schichtdicke ist 2 mm. Zur Analyse der pQCT-Schichtbilder wird die integrierte Software (Version 6.20C) verwendet. Die extrahierten FK wurden in einem modifizierten Transportbehälter (Fa. Telos) reproduzierbar anhand einer studienspezifischen SOP gelagert. Anschließend erfolgt die Messung des gesamten FK-Volumens in zwei unterschiedlichen Schichtungen:

- Gesamtvolumen in kontinuierlicher Schichtung, Inkrement 2 mm
- Teilvolumen in partieller Schichtung, Inkrement 11 mm

Der FK besitzt eine deutlich dünnere Kortikalis als die Standardmessorte am distalen Radius und an der distalen Tibia. Beeinflusst durch den Partialvolumeneffekt ist somit eine korrekte Erfassung des Analysevolumens am FK mit den Standardeinstellungen nicht möglich. Deshalb wurden die Analyseparameter für diese Studie entsprechend ausführlicher Voruntersuchungen angepasst. Die gesamte (tot) und die trabekuläre (trab) BMD, BMC und CSA wurden jeweils mit den Schwellenwerten ThBd 180 mg/ccm, ThBd2 400 mg/ccm, ContMode 1, PeelMode 2 und Filters 2 F03F05 berechnet. Die kortikale (kort) BMD, BMC und CSA wurden jeweils mit dem Schwellenwert ThCort 400 mg/ccm und CortMode 1 berechnet.

Für beide Schichtungen wurden die Ergebnisse der Einzelschichten zu Gesamtscores zusammengefasst und diese (Normalbefund vs. pathologische Befunde) mit den Ergebnissen des Tabea-FK-Scores verglichen. Eine niedrige trabekuläre BMD ist richtungsweisend für eine Osteoporose. Bestehen Veränderungen der ursprünglich trabekulären Region durch degenerative Umbauprozesse, wie z. B. Sklerosierung und zystische Veränderungen, verändern sich die Verhältnisse der Quotienten aus trab, kort und tot BMC, wie auch die Quotienten aus trab, kort und tot CSA. Die Analysen wurden elektronisch gespeichert und ein Messbericht erstellt, wobei die Messung in Form einer Mittelschicht dokumentiert wurde und einzelne Messparameter berichtet wurden.

### Histologie und makroskopische Zweitbewertung

Die präoperative Evaluation der FK anhand der standardisierten Röntgenbilder und der intraoperativen Bewertung sollte auf ihre Aussagefähigkeit überprüft werden. Dazu wurden sie in der Universitäts-Gewebebank, Institut für Transfusionsmedizin Charité – Universitätsmedizin Berlin, nach einem festgelegten Standard präpariert. Hierbei wurde primär ein Zylinder (Länge 15 mm, Durchmesser 5 mm) aus dem peripheren oberen Anteil des FK gewonnen (Abb. 4).

Der Zylinder wurde in 4 % gepuffertem Formalin gelagert und danach mit EDTA entkalkt. Nach Fixierung, Zuschnitt und Färbung erfolgte die histologische Beurteilung (Kortikalis, Spongiosa, Zysten, Nekrosen, Verkalkungen) ebenfalls in einer am Primärscore angelehnten Bewertung (Kl. 1, 2, 3).

**Figure 5:**
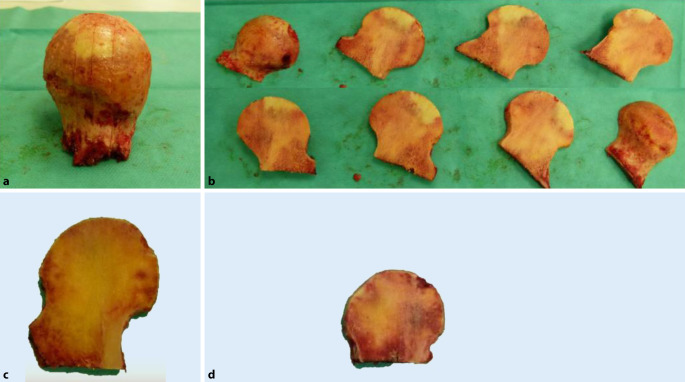

Darüber hinaus erfolgte durch zwei der ursprünglich entnehmenden Orthopäden eine Kontrolle der Größenangaben und eine nochmalige, jetzt makroskopische Quantifizierung (Makroscore, Bewertung siehe Abb. 5) des in vier Schichten aufgesägten FK. Dieses Verfahren erlaubt anhand der makroskopisch-morphologischen Beurteilung eine sichere Beurteilung der Anzahl der Zysten, einer eventuell vorhandenen Deformität sowie einer Osteoporose. Ebenso ist die Größe zweifelsfrei bestimmbar. Diese makroskopisch-morphologische Beurteilung und die daraus folgende Einteilung in den Makroscore kann daher als Goldstandard der Qualitätsbeurteilung angesehen werden. Die übrigen Scores wurden mit diesem Makroscore verglichen. Die Bewertung des Makroscores erfolgte verblindet und ohne Kenntnis der Scores aus den klinisch-radiologischen und histologischen Bewertungen.

### Statistik

Die Übereinstimmung der Qualitätsbeurteilung des aufgeschnittenen FK (Makroscore, Goldstandard) mit dem klinisch-radiologischen Tabea-FK-Score und dem Histo-Score bzw. dem pQCT-Score wurde mittels der Berechnung der Sensitivitäten und Spezifitäten quantifiziert. Diese wurden für die mittlere versus beste Qualität (Scorewerte 2 und 1) sowie die schlechte und mittlere Qualität (Scorewerte 3 und 2) separat berechnet. Als letztes wurden die der mittleren und besten Qualität kombiniert (Scorewerte 1 und 2) versus Scorewert 3 (schlechte Qualität) und die Sensitivitäten und Spezifitäten der verschiedenen Vergleiche berechnet.

Folgende Vergleiche wurden erstellt:

- Tabea-FK-Score vs. Makroscore
- Histo-Score vs. Makroscore
- pQCT-Score vs. Makroscore

## Ergebnisse

**Figure 6:**
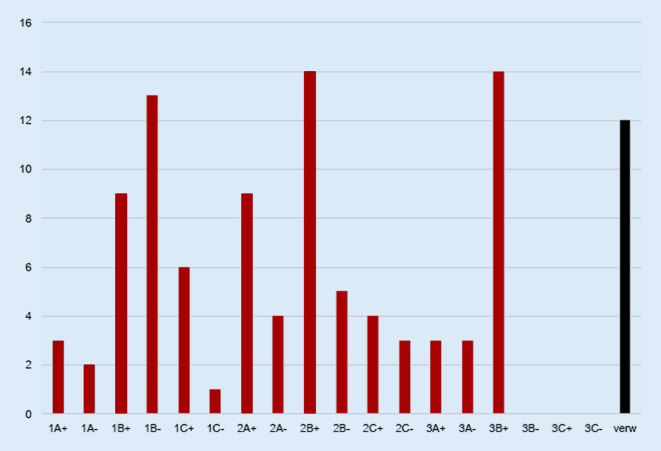

**Figure 7:**
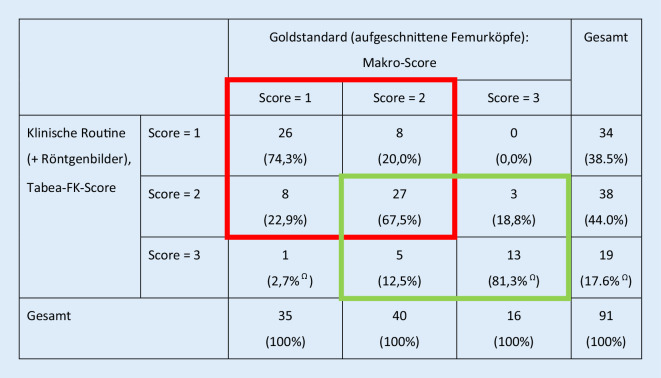

In Abb. 6 sind die Verteilungen der 105 FK auf die Endscores dargestellt. Von diesen 105 Präparaten wurden primär 12 verworfen, da sie nicht für eine FK-Transplantation geeignet schienen. Als interessante Nebenbefunde wurden in einem Fall (TAB-070) in der histologischen Untersuchung Zellen eines sehr kleinen Osteoblastoms (<2 mm) und in einem zweiten Fall (TAB-043) Zellen eines Enchondroms nachgewiesen. Diese beiden Präparate wurden aus der Studie ausgeschlossen. In Abb. 7 ist die Übereinstimmung des Makroscores mit dem klinisch-radiologischen Tabea-FK-Score dargestellt. Die höchste Übereinstimmung fand sich in Bezug auf die FK mit schlechter Qualität (81 %) und die geringste in Bezug auf die FK mit mittlerer Qualität (68 %). Bezogen auf die Unterscheidung zwischen FK mit bester und mittlerer Qualität fand sich eine Sensitivität des Tabea-FK-Scores in Bezug auf den Goldstandard von 77 % (95 %-Konfidenzintervall, 95 %-KI: 60–90 %) und eine Spezifität von 76 % (95 %-KI: 59–89 %). In Bezug auf die Unterscheidung von FK von mittlerer und schlechter Qualität fand sich eine Sensitivität von 81 % (95 %-KI: 54–96 %) und eine Spezifität von 84 % (95 %-KI: 67–95 %).

Sollen FK mit schlechter Qualität von denjenigen mit bester oder mittlerer Qualität unterschieden werden, zeigt die radiologisch-klinische Beurteilung (Tabea-FK-Score) eine Sensitivität von 81 % und eine Spezifität von 92 % (Tab. 3, Spalten 6–7). Wie die Qualitätsbeurteilung mittels des Histo-Scores (Tab. 1) sowie mittels des pQCT-Scores (Tab. 2) im Vergleich mit dem Makroscore zeigen (die Sensitivitäten und Spezifitäten zeigt Tab. 3), sind die Sensitivitäten bezüglich der Unterscheidung von FK bester und mittlerer Qualität noch als gut einzuschätzen (>80 %), jedoch zeigten sich bei der Unterscheidung von FK schlechter und mittlerer Qualität deutlich geringere Sensitivitäten (54 % und lediglich 9 %). Dies zeigt sich ebenfalls, wenn es darum geht, FK schlechter Qualität von denjenigen mit mittlerer und bester Qualität abzugrenzen. Hier zeigt der Histo-Score lediglich eine Sensitivität von 44 % und der pQCT-Score eine Sensitivität von 6 %.

**Table 1:** 

		Makroscore = Goldstandard (makroskopische Beurteilung der aufgeschnittenen Femurköpfe)			Gesamt
		Score = 1	Score = 2	Score = 3	
Histo-Score (histologische Beurteilung eines Standardzylinders)	Score = 1	23 (65,7 %)	6 (15,0 %)	3 (18,8 %)	32 (35,2 %)
	Score = 2	12 (34,3 %)	33 (82,5 %)	6 (37,5 %)	51 (56,0 %)
	Score = 3	0 (0,0 %)	1 (2,5 %)	7 (43,8 % ^Ω^)	8 (8,8 %)
Gesamt		35 (100 %)	40 (100 %)	16 (100 %)	91 (100 %)

*Score 1* beste Qualität, *Score 2* mittlere Qualität, *Score 3* schlechte Qualität, *Ω *summiert sich nicht auf 100 % wegen Rundungsfehlern

Die Beurteilung des biologischen Materials (aufgeschnittener Femurkopf = Goldstandard) erfolgte anhand der in Abb. 1 gezeigten „Checkliste humaner Femurkopf“

**Table 2:** 

		Makroscore = Goldstandard (makroskopische Beurteilung der aufgeschnittenen Femurköpfe)			Gesamt
		Score = 1	Score = 2	Score = 3	
pQCT-Score	Score = 1	9 (25,7 %)	7 (17,5 %)	5 (31,3 %)	32 (35,2 %)
	Score = 2	22 (62,9 %)	32 (80,0 %)	10 (62,5 %)	51 (56,0 %)
	Score = 3	4 (11,4 %)	1 (2,5 %)	1 (6,3 % ^Ω^)	8 (8,8 %)
Gesamt		35 (100 %)	40 (100 %)	16 (100 %)	91 (100 %)

*Score 1* beste Qualität, *Score 2* mittlere Qualität, *Score 3* schlechte Qualität, *Ω *summiert sich nicht auf 100 % wegen Rundungsfehlern, *pQCT* periphere quantitative Computertomographie

Die Beurteilung des biologischen Materials (aufgeschnittener Femurkopf = Goldstandard) erfolgte anhand der in Abb. 1 gezeigten „Checkliste humaner Femurkopf“

**Table 3:** 

	Score 2 versus Score 1		Score 3 versus Score 2		Score 3 versus Score 1 + 2	
	Sensitivität	Spezifität	Sensitivität	Spezifität	Sensitivität	Spezifität
Tabea-FK-Score	0,77 (0,60–0,90)	0,76 (0,59–0,89)	0,81 (0,54–0,96)	0,84 (0,67–0,85)	0,81 (0,54–0,96)	0,92 (0,83–0,97)
Histo-Score	0,85 (0,69–0,94)	0,66 (0,48–0,81)	0,54 (0,25–0,81)	0,97 (0,85–1,00)	0,44 (0,20–0,70)	0,99 (0,93–1,00)
pQCT-Score	0,82 (0,66–0,92)	0,29 (0,14–0,48)	0,09 (0,00–0,41)	0,97 (0,84–1,00)	0,06 (0,00–0,30)	0,93 (0,85–0,98)

*Score 1* beste Qualität, *Score 2* mittlere Qualität, *Score 3* schlechte Qualität, *pQCT* periphere quantitative Computertomographie

## Diskussion

Wissenschaftliche Untersuchungen zur klinisch-morphologischen Bewertung des FK zur Transplantation liegen derzeit nicht vor. In der Literatur sind vor allem Studien und Untersuchungen zu mikrobiologischen [9, 19, 22, 23], immunologischen [13, 26], infektionssicherheitsrelevanten [10, 14, 17, 24] und ökonomisch-pharmazeutischen Fragestellungen [1, 12, 15, 16] zu finden.

Die makroskopische Untersuchung in der vorliegenden Studie an in vier gleich dicke Scheiben geschnittenen FK ergab eine hohe Übereinstimmung mit der radiologischen/klinischen Klassifikation. Daher kann aus unserer Sicht die radiologisch/klinische Beurteilung als valide gelten und auf die zerstörende makroskopische Untersuchung in der klinischen Routine verzichtet werden. Die präoperative radiologische Analyse erkennt zuverlässig das Ausmaß zystischer oder nekrotischer/sklerotischer Läsionen, welche den FK als Transplantat ungeeignet erscheinen ließen. Auch Deformitäten sind in ihrem Ausmaß eindeutig zu detektieren. Das Volumen des Kopfes ist durch den Querdurchmesser an der breitesten Stelle definiert. Die Binnenstruktur ist als native Spongiosa anzusehen, abzüglich des Ausmaßes an Zysten und Sklerosen. Dieses Ausmaß beschreibt der Score.

Um den Score einfach handhabbar und somit für den klinischen Alltag tauglich zu machen, wurde jede einzelne Kategorie in drei Grade eingeteilt. Eine genauere Klassifikation ist daher nicht erforderlich.

In der vorgelegten Studie wurde anhand eines klinisch-radiologischen Scores versucht, die Beurteilung der FK-Qualität durch den entnehmenden Arzt bereits intraoperativ zu standardisieren. Die FK wurden anschließend mittels pQCT auf das Anteilsverhältnis von spongiösem und kortikalem Knochen untersucht und Knochendichte, -masse und die Masseverteilung bildlich dargestellt. Mit dieser hoch innovativen Methode können sowohl die BMD, als auch die BMC quantifiziert werden. Eine gute Übereinstimmung mit dem makroskopisch-morphologischen Status (hier mittels des Makroscores abgebildet) hätte einen neuen Weg in der Standardisierung von Knochentransplantaten eröffnet.

Etwas überraschend war daher die schwache Übereinstimmung zwischen dem Makroscore und dem pQCT-Score. Ursächlich hierfür könnte sein, dass die gewählten Vergleichscores das klinische Korrelat im FK nicht widerspiegeln. Erhart et al. konnten in einer vergleichenden Untersuchung der CT-gestützten Messung der Spongiosaknochendichte bei proximalen Femurfrakturen gute Ergebnisse erzielen [6]. Den Autoren gelang eine Abgrenzung osteoporotischer Veränderungen zum Normalbefund, die die Individualisierung der Therapieansätze erleichterte. Eine weitere diagnostische Möglichkeit zur Messung der Knochendichte zeigen Craiovan et al., welche die Dual Energy X‑Ray Absorptiometry (DXA) zur Unterscheidung von Knochendefekten infolge von FK-Nekrose oder primärer Osteoarthritis anwendeten [5]. Das in der hier vorgelegten Studie verwendete pQCT ist derzeit als qualitatives und quantitatives Kriterium für die Bewertung der knöchernen Anteile des FK nicht zu empfehlen, sollte jedoch methodisch weiterentwickelt werden.

Die Feststellung, dass die Histologie nur eine mittelmäßige Übereinstimmung mit dem makroskopisch-morphologischen Status aufweist, war aufgrund des ausgewählten Volumens des Untersuchungsmaterials nicht unerwartet, da der entnommene Standardzylinder nur eine kleine Region des FK und nicht dessen gesamte Struktur widerspiegelt. Trotzdem ist die Histologie eine sinnvolle Ergänzung der Qualitätsbewertung, insbesondere dann, wenn das Probevolumen erhöht wird. Schließlich sollte auch bedacht werden, dass die histologische Untersuchung in Einzelfällen wertvolle Hinweise auf pathologische Veränderungen aufzeigen kann. Sugihara et al. fanden in einer histologischen Untersuchung von nicht verwendeten FK in 5 von 89 Fällen auffällige Befunde (3 Fälle mit Verdacht auf B‑Zell-Lymphom, 1 Fall mit Verdacht auf monoklonale Plasmozytosis und 1 Fall mit unspezifischer Entzündung des Knochenmarks) [21]. Zu ähnlichen Ergebnissen gelangen Zwitser et al. Die in 14/853 Femurköpfen Hinweise auf ein B‑Zell-Lymphom fanden [26]. Auch in unserer Untersuchung wurden in zwei Fällen pathologische Veränderungen (Enchondrom, sehr kleines Osteoblastom) im Rahmen der Histologie nachgewiesen.

Alle strukturellen Transplantate müssen intraoperativ geformt werden, um die knöcherne Defektstrecke bündig zu ersetzen. Um dem Operateur diese technisch anspruchsvolle Aufgabe zu erleichtern, sollten keine FK mit zystischen Läsionen oder Sklerosen/Nekrosen verwendet werden. Die Größe des Kopfes muss im Vergleich zur Größe des zu ersetzenden Defektes gewählt werden, sofern es sich um ein strukturelles Transplantat handelt. Eine Osteoporose sollte für ein strukturelles Transplantat gar nicht oder allenfalls wenig ausgeprägt sein. Der vollständig ausgefüllte Score sollte dem Transplantat beiliegen, um geeignete Köpfe identifizieren zu können. Dies könnte auch bereits in der Knochenbank erfolgen. Alle anderen Köpfe sind nur als „morcellized graft“ verwendbar.

Die Bewertung der Kriterien des primären FK-Scores wurde im Rahmen einer vergleichbaren makroskopischen Bewertung am aufgesägten FK durch dieselben Operateure verblindet wiederholt. Die entsprechende Sensitivität und Spezifität der Beurteilung bester + mittlerer versus schlechter Qualität wurde in der durchgeführten statistischen Analyse als gut bewertet. Dies unterstreicht die Fähigkeiten langjährig operativ tätiger Orthopäden, die Qualität der Knochenspongiosa bereits anhand des Röntgenbildes und des intraoperativen Befundes korrekt einzustufen. Insofern kann die Einführung des Tabea-FK-Scores in die Routineabläufe von Knochenbanken empfohlen werden. Mittels des Scores werden dem Kliniker verlässliche Hinweise in Bezug auf die Knochengewebequalität gegeben, die ihm auch in Bezug auf die Transplantatauswahl indikationsbezogen unterstützen. Weiterführende nicht zuletzt auch kostenintensive diagnostische Maßnahmen wie pQCT oder Histologie, sind nicht erforderlich und für den verlässlichen Befund derzeit auch nicht geeignet.

Die Einführung des Tabea-FK-Scores wird daher insbesondere für lokale Knochenbanken als qualitätssichernde Maßnahme empfohlen. Es sollte jedoch beachtet werden, dass eine umfangreiche Schulung und interaktive Kommunikation zwischen den Operateuren erfolgt, um die Bewertungskriterien innerhalb einer Knochenbank zu standardisieren.

## Fazit für die Praxis

- Die Qualität und Eignung eines für Transplantationszwecke entnommenen Femurkopfes (FK) kann durch erfahrene Endoprothetiker bereits präoperativ und auf klinisch-radiologischer Basis verlässlich eingeschätzt werden.
- Für die standardisierte Erfassung bietet sich ein Score an, der neben den klinisch-radiologischen Parametern (Zysten, Nekrosen, Verkalkungen, Deformitäten, Osteoporose) auch Größe und Schenkelhalsanteil erfasst.
- Die Anwendung des vorgeschlagenen Tabea-FK-Scores bietet Knochenbanken ein Qualitätswerkzeug zur gezielten Auswahl von FK-Transplantaten für die jeweilig vorgesehene Operationsindikation („morcellized“ vs. strukturelles Graft).

## Abkürzungen

AMG: Arzneimittelgesetz
BMC: „Bone mineral content“
BMD: „Bone mineral density“
CSA: Querschnittsfläche
DXA: Dual Energy X‑Ray Absorptiometry
EDTA: Ethylendiamintetraacetat
FK: Femurkopf
KI: Konfidenzintervall
kort: Kortikal
pQCT: Periphere quantitative Computertomographie
SEC: Single European Code
SOP: Standard Operating Procedure
TEP: Totalendoprothese
tot: Gesamt
TPG: Transplantationsgesetz
TPG-GewV: TPG-Gewebeverordnung
trab: Trabekulär
